# Genetic Architecture of Growth Traits in Turbot (*Scophthalmus maximus*) Revealed by Genome-Wide Association Studies

**DOI:** 10.3390/ijms27156689

**Published:** 2026-07-27

**Authors:** Ao Chen, Shuo Liang, Li Jiang

**Affiliations:** 1 National Demonstration Center for Experimental Fisheries Science Education, Shanghai Ocean University, Shanghai 201306, China; m19503860141@163.com (A.C.); 13722204240@163.com (S.L.); 2 Research Centre for Aquatic Biotechnology, Chinese Academy of Fishery Sciences, Beijing 100141, China; 3 Beijing Key Laboratory of Fishery Biotechnology, Chinese Academy of Fishery Sciences, Beijing 100141, China; 4 Key Laboratory of Aquatic Genomics, Ministry of Agriculture and Rural Affairs, Beijing 100125, China

**Keywords:** turbot (*Scophthalmus maximus*), genome-wide association study, growth traits, pleiotropic loci, heritability, marker-assisted selection

## Abstract

Turbot (*Scophthalmus maximus*) is a key farmed flatfish species in North China, where growth performance directly determines aquaculture profitability. To elucidate the genetic architecture underlying growth traits, we performed whole-genome resequencing of 177 turbot individuals and obtained 5.29 million high-quality single nucleotide polymorphisms (SNPs). Genome-wide association studies (GWAS) were conducted for eleven growth traits—body weight (BW), total length (TL), body length (BL), body depth (BD), trunk length (TUL), head length (HL), snout length (SnL), caudal peduncle depth (CPD), eye diameter (ED), post-orbital head length (PoL), and interorbital width (IW)—using both single-trait and multi-trait approaches. Heritability estimates ranged from 0.001 (IW) to 0.259 (BW), with BW showing the highest heritability, and genetic correlation analysis revealed strong positive correlations between BW and most body size traits (*r*_g_ > 0.7). Critically, extensive genetic pleiotropy governing these traits was uncovered. In single-trait GWAS, two pleiotropic loci—19:16017254 (within *fam81b*) and 15:2921091 (within *atrn*)—were repeatedly associated with the same six body-size traits (BD, BL, CPD, HL, TL, and TUL), demonstrating that single variants can exert coordinated effects across multiple morphological dimensions. Furthermore, multi-trait GWAS captured additional pleiotropic signals that remained undetected in single-trait analyses, identifying 20 novel loci encompassing key candidates such as *iqgap1* (cytoskeletal signaling), *enah* (actin organization)*, and baz2a (chromatin remodeling)*. KEGG enrichment further consolidated these findings, with candidate genes significantly enriched in the “Regulation of actin cytoskeleton” pathway, while *amd1* emerged as a hub gene integrating three metabolic pathways. Collectively, these results highlight the pervasive pleiotropy underlying turbot growth and serve as promising candidate loci for marker-assisted selection, although independent validation in larger populations is required before practical application.

## 1. Introduction

Turbot (*Scophthalmus maximus*) is a commercially valuable flatfish species that has been farmed and fished for decades. Overfishing and anthropogenic pressures have sharply reduced wild stocks, while market demand continues to rise [1]. Aquaculture has increasingly supplied this demand, and over the past 20 years, farmed turbot production in Europe and China has grown substantially, reaching 59,615 tons [2]. However, this increase has been driven primarily by industry expansion rather than by improvements in individual growth performance. Selective breeding programs based on microsatellite markers have been initiated to enhance growth rates, achieving a 65% gain in body weight within five generations [3,4,5]; nevertheless, the underlying genetic architecture of growth traits remains incompletely characterized.

Growth traits are key economic determinants in aquaculture, directly influencing production efficiency, time to market, and profitability [6,7]. Growth is a complex polygenic trait associated with numerous genes involved in metabolism, hormone regulation, and development. Well-characterized components of the endocrine growth axis—including growth hormone (GH), its receptor (GHR), insulin-like growth factors (IGFs), and their regulatory peptides—play established roles in growth regulation [8,9,10,11]. Additionally, genes controlling muscle tissue development, such as myostatin (*MSTN*), myogenic regulatory factors (*MRFs*), and myomaker (*MYMK*), are critically involved in muscle growth. Notably, MSTN knockout has been shown to promote growth in several fish species, including olive flounder (*Paralichthys olivaceus*) [12], channel catfish (*Ictalurus punctatus*) [13], common carp (*Cyprinus carpio*) [14], and red sea bream (*Pagrus major*) [15]. Despite these advances, the full repertoire of growth-related genes and their regulatory networks in turbot has yet to be systematically elucidated.

Genome-wide association study (GWAS) is a powerful statistical approach that employs dense genome-wide markers to identify genetic loci associated with complex traits [16]. By leveraging linkage disequilibrium (LD), GWAS maps single nucleotide polymorphisms (SNPs) to phenotypic variation [17,18,19]. This approach has been widely applied to dissect growth, disease resistance, and stress tolerance traits in diverse aquaculture species, including olive flounder [20], large yellow croaker (*Larimichthys crocea*) [21], rainbow trout (*Oncorhynchus mykiss*) [22], Atlantic salmon (*Salmo salar*) [23], common carp [24], and ayu (*Plecoglossus altivelis*) [25]. While single-trait GWAS has proven effective in identifying trait-specific loci, it often misses variants with pleiotropic effects that influence multiple correlated traits. Multi-trait GWAS, by jointly analyzing multiple phenotypes, can uncover such shared genetic architecture and improve statistical power for detecting pleiotropic loci [26].

In this study, we performed whole-genome resequencing of 177 turbot individuals and generated 5.29 million high-quality SNPs. We conducted both single-trait and multi-trait GWAS for eleven growth traits—body weight (BW), total length (TL), body length (BL), body depth (BD), trunk length (TUL), head length (HL), snout length (SnL), caudal peduncle depth (CPD), eye diameter (ED), post-orbital head length (PoL), and interorbital width (IW). Our analytical framework integrated heritability estimation, genetic correlation analysis, population structure assessment, and KEGG pathway enrichment to comprehensively dissect the genetic basis of growth. Through this integrated strategy, we aimed to (i) elucidate the genetic architecture of growth traits in turbot, (ii) identify pleiotropic loci and candidate genes with potential for MAS, and (iii) provide a foundation for genomic selection in turbot breeding programs.

## 2. Results

### 2.1. Genetic Correlations Among Traits

Table 1 presents the genetic correlation coefficients among 11 growth traits in turbot. Body weight (BW) showed strong positive correlations with most body size traits, highest with body depth (BD, *r*_g_ = 0.864), followed by total length (TL, 0.842), body length (BL, 0.766), and caudal peduncle depth (CPD, 0.730). TL was highly correlated with BL (0.872), BD (0.872), and trunk length (TUL, 0.837). Among head traits, head length (HL) and post-orbital head length (PoL) were strongly correlated (0.776), whereas eye diameter (ED) showed weak or negative correlations with nearly all traits (e.g., −0.047 with BW, −0.232 with snout length), suggesting independent genetic regulation. CPD also correlated moderately with BD (0.713) and TUL (0.585), indicating its potential as an indirect selection indicator for BW. Statistical significance of the genetic correlations was assessed using likelihood ratio tests, with *p*-values adjusted for multiple testing using the Benjamini–Hochberg procedure (false discovery rate < 0.05).

**Figure 1:**
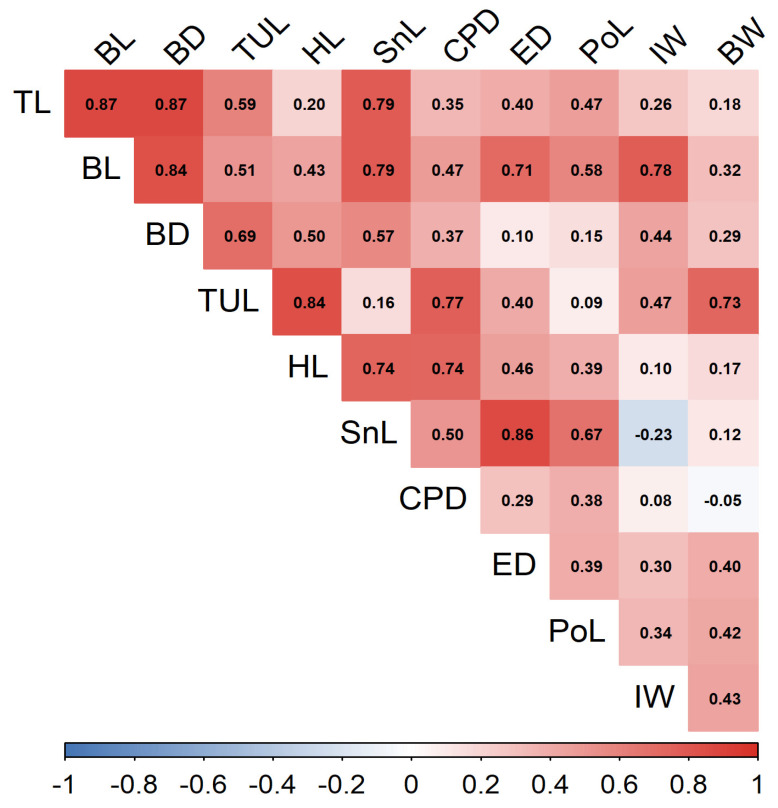
Genetic correlation matrix of the 11 growth traits in turbot (*Scophthalmus maximus*). The color gradient of blue to red shows the magnitude and direction of genetic correlation (*r*_g_).

Figure 1 visualizes the correlation matrix as a heatmap, with blue-to-red gradient representing the magnitude and direction of *r*_g_. Deep blue blocks (*r*_g_ > 0.7) cluster prominently among core body size traits (TL, BL, BD, TUL, and CPD) and also among head traits (HL, PoL, and IW). Body weight is centrally located and strongly connected to the body-size cluster, while eye diameter appears isolated with pale or light-colored blocks, reinforcing its weak genetic association with other traits. This visual pattern confirms the numerical findings and provides an intuitive overview of the genetic architecture underlying growth traits in turbot.

### 2.2. Heritability Estimates

**Figure 2:**
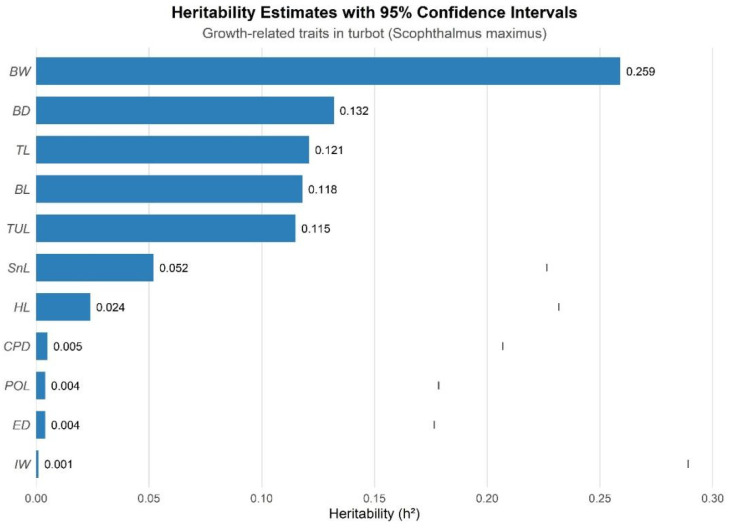
Heritability estimates (h^2^) of the 11 growth-related traits in turbot (*Scophthalmus maximus*). The error bars are standard errors (Se), and the dashed lines indicate the confidence intervals for the heritability estimates.

The heritability estimates (h^2^) of IW and BW were 0.001 and 0.259 respectively (Table 2, Figure 2). BW had the highest heritability (0.259 ± 0.158) and was considered moderate. The traits of body size (BD, TL, BL, and TUL) had low heritability (0.115–0.132). Head and eye traits (SnL, HL, CPD, PoL, ED, and IW) had very low heritability (≤0.05) and thus had little additive genetic variation and were strongly influenced by the environment. Although the heritability was small, both ED and SnL had relatively large phenotypic coefficients of variation (24.87% and 18.72%), and this was due to a large number of environmental factors.

### 2.3. Population Genetic Structure

**Figure 3:**
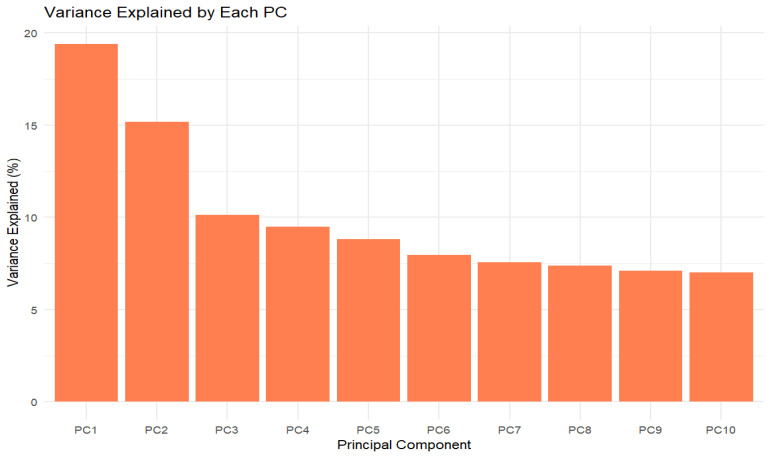
Scree plot of principal component analysis based on 5.29 million high-quality SNPs.

To characterize the genetic background of the sampled population, we performed principal component analysis (PCA) based on the 5.29 million high-quality SNPs. The first three principal components (PCs) accounted for 44.68% of the total genetic variance, with PC1, PC2, and PC3 explaining 19.39%, 15.17%, and 10.12%, respectively (Table 3; Figure 3). The first ten PCs collectively captured 100% of the variance, indicating that the SNP data comprehensively represented the genetic structure of the cohort.

Genetic diversity indices were estimated for the 177 individuals. The mean observed heterozygosity (Ho) was 0.1818, while the mean expected heterozygosity (He) was 0.1979, yielding an average inbreeding coefficient (F) of 0.0813 (Table 4). Ho values ranged from 0.0934 to 0.2229, and F ranged from −0.126 to 0.0813. These moderate-to-low diversity estimates are consistent with those typically observed in selectively bred aquaculture populations and suggest that the cohort was sufficiently homogeneous for reliable association mapping. Collectively, the PCA and diversity metrics confirmed the absence of strong population stratification, supporting the robustness of subsequent GWAS analyses.

### 2.4. Results of Single-Trait GWAS

**Figure 4:**
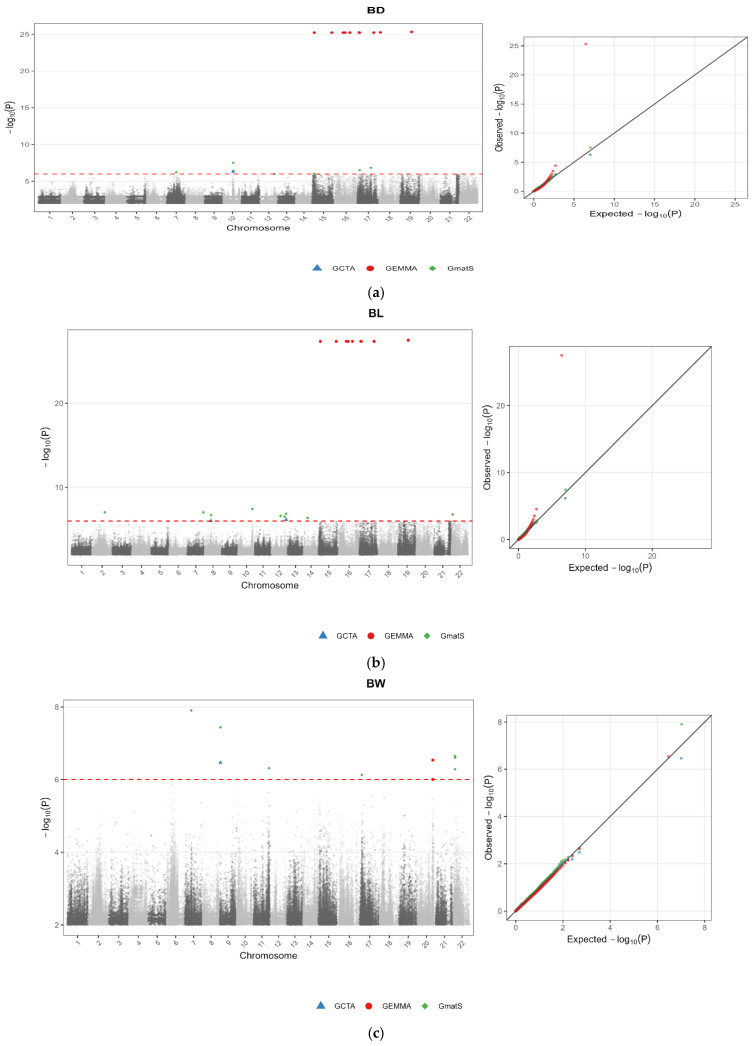

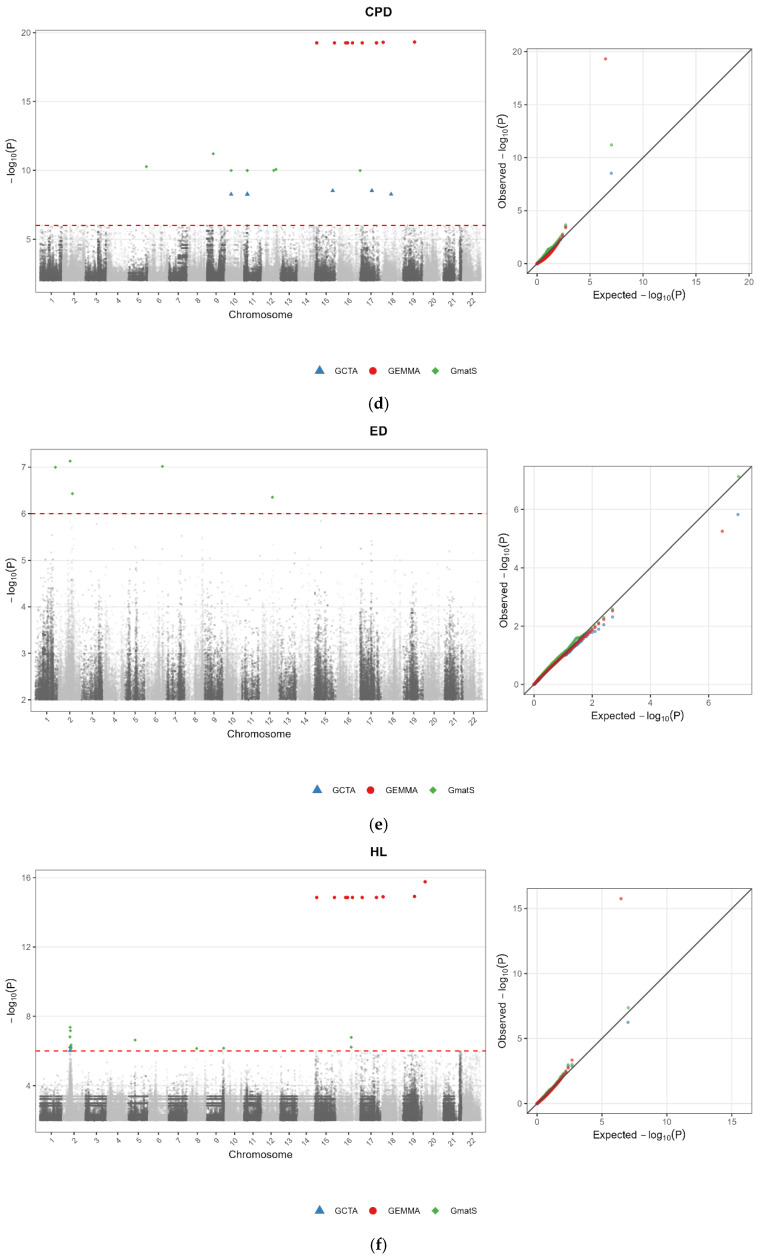

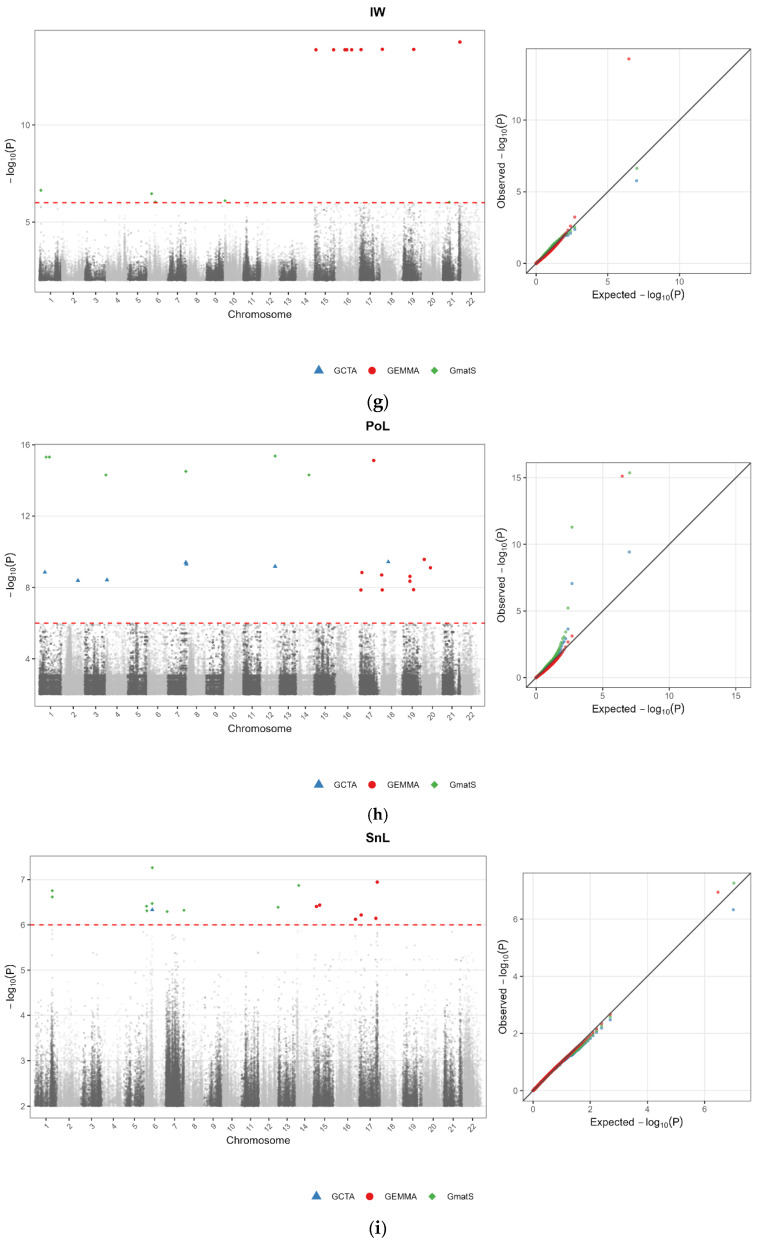

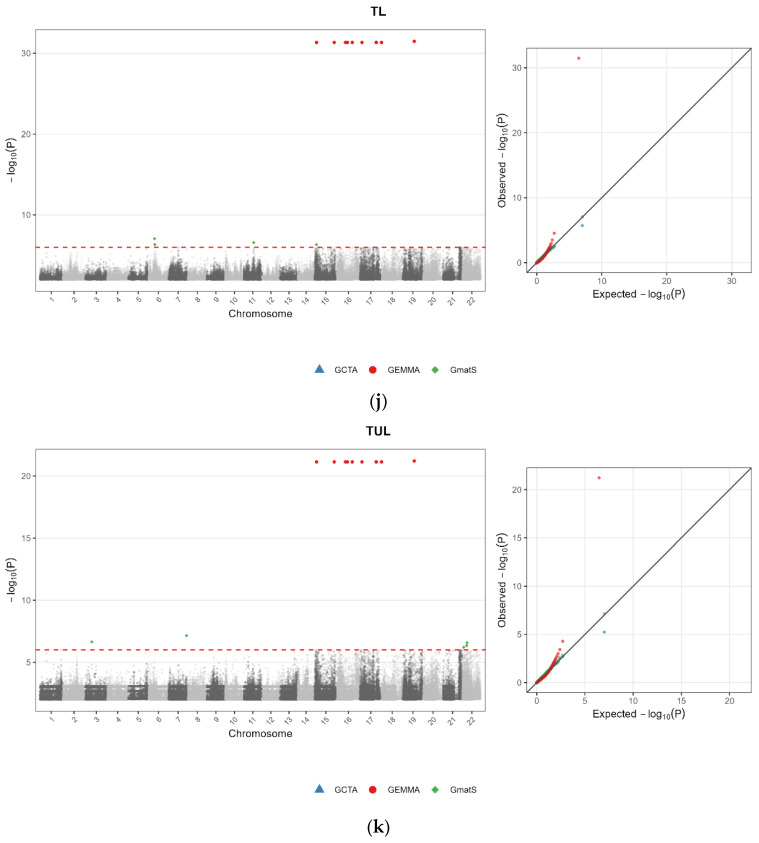
Manhattan plots (**left**) and Q-Q plots (**right**) for the single-trait GWAS results of the 11 growth-related traits in turbot (*Scophthalmus maximus*) obtained by GEMMA, GCTA and GmatS. SNPs above the horizontal line are significant in the association (*p* < 1 × 10^−6^). (**a**) body depth (BD), (**b**) body length (BL), (**c**) body weight (BW), (**d**) caudal peduncle depth (CPD), (**e**) eye diameter (ED), (**f**) head length (HL), (**g**) interorbital width (IW), (**h**) post-orbital head length (Pol), (**i**) snout length (SnL), (**j**) total length (TL), (**k**) trunk length (TUL).

Single-trait GWAS for the 11 growth traits was performed using three software packages—GEMMA, GCTA, and GmatS—with a genome-wide significance threshold of *p* < 1 × 10^−6^. Manhattan and Q-Q plots for each trait are presented in Figure 4a–k. The Q-Q plots showed that observed *p*-values closely followed the expected null distribution in the lower tail, with clear deviations in the upper tail, indicating genuine association signals. Genomic inflation factors were approximately 1 for all traits, confirming that population stratification was adequately controlled.

In total, 55 significant SNPs (*p* < 1 × 10^−6^) were identified across the 11 traits (top five SNPs per trait; Table 5), distributed on all chromosomes except 8, 10, 13, and 14. The major growth traits (BW, TL, BL, and BD) all exhibited strong association signals; for example, the most significant SNP for TL (19:16017254) reached *p* = 3.35 × 10^−32^ with an effect size of −6.87. Among the three software packages, GEMMA identified the most significant SNPs (45), followed by GmatS (10), while GCTA detected none among the top five, highlighting differences in algorithmic sensitivity.

Several genomic regions exhibited pleiotropic associations across multiple traits. A SNP at position 16,017,254 bp on chromosome 19 was among the most significant markers for six traits (BD, BL, CPD, HL, TL, and TUL), with the nearest gene being *fam81b*, which is putatively involved in cell cycle regulation. Similarly, a SNP at 2,921,091 bp on chromosome 15, located within the *atrn* gene (melanocortin signaling pathway), was associated with the same six traits. To assess whether these two loci represent independent pleiotropic signals, we calculated pairwise LD (r^2^) between the lead SNPs using PLINK. The r^2^ between chr19:16017254 and chr15:2921091 was 0.012, indicating that these two loci are effectively independent. These findings suggest the presence of pleiotropic QTLs governing multiple growth traits.

Some SNPs resided within gene bodies (distance = 0), including *amd1* (polyamine biosynthesis), *dlgap3* (synaptic signaling), and *slc6a1l* (GABA transport). Others were located in intergenic regions but in proximity to potential regulatory genes (e.g., SNP 17:2415956, ~20.7 kb from *eve1*), suggesting possible long-range regulatory effects. Effect size directions varied across traits, likely reflecting the complex genetic architecture of growth or differences in statistical modeling among software packages.

### 2.5. Multi-Trait GWAS Results

To identify genetic loci affecting multiple traits, multi-trait GWAS was performed for four trait combinations using GEMMA_mv, GmatS_mv, and the R package MultiTrait, with a significance threshold of *p* < 1 × 10^−6^. Importantly, after excluding SNPs that had reached significance in any single-trait GWAS (*p* < 1 × 10^−6^), multi-trait analysis uncovered 20 novel loci that were not detected in any single-trait analysis (Table 6). These loci, distributed across chromosomes 1, 3, 4, 5, 6, 9, 13, 14, 16, 18, 19, 20, and 21, represent pleiotropic signals that emerged exclusively through joint analysis of multiple traits.

**Figure 5:**
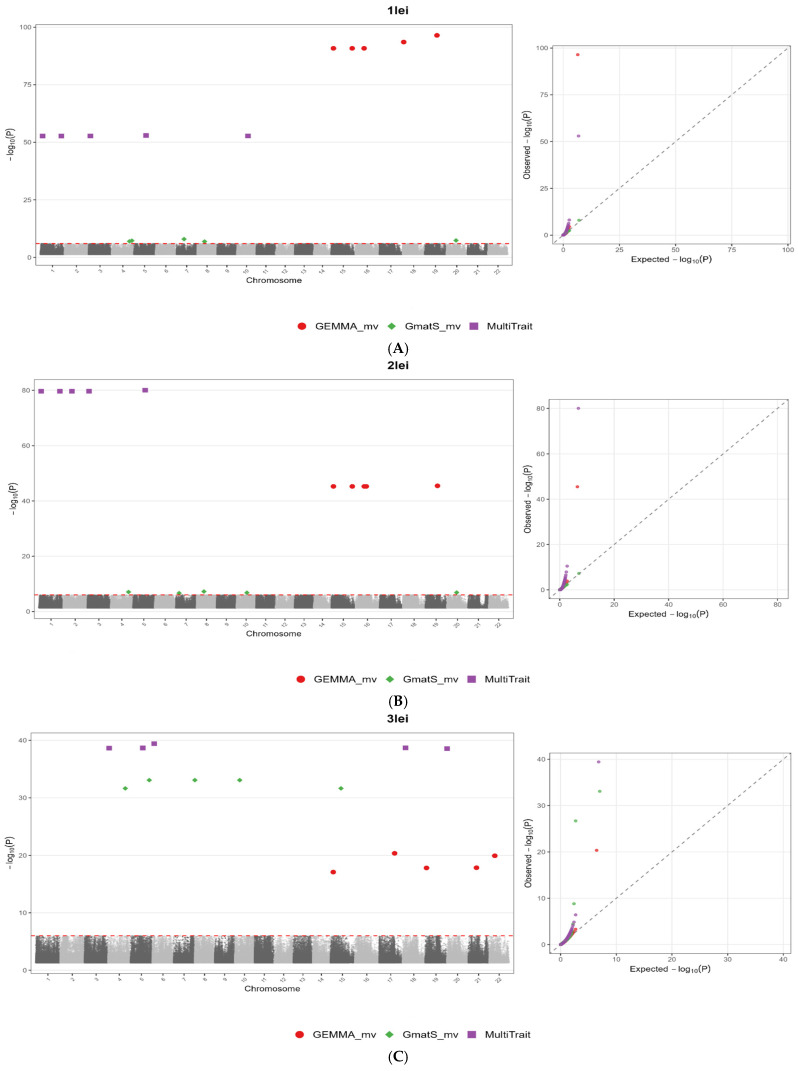

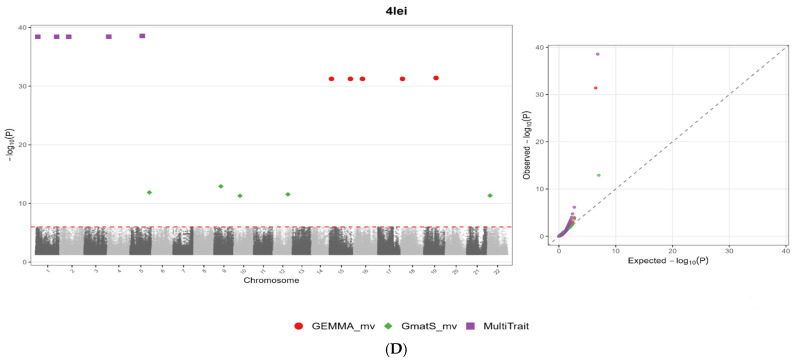
Multi-trait GWAS results for the four-trait combination in Turbot (*Scophthalmus maximus*). Manhattan plots (**left**) and Q-Q plots (**right**) are shown for (**A**) trait combination 1 (BW), (**B**) combination 2 (TL, BL, BD), (**C**) combination 3 (HL, SnL, ED, PoL, IW), and (**D**) combination 4 (TUL, CPD). The red dashed line is the significance threshold (*p* = 1 × 10^−6^). Colorful SNPs are presented in different colors by software: GEMMA_mv (red circles), GmatS_mv (green diamonds), MultiTrait (purple squares).

For trait combination 1 (BW), GEMMA_mv identified five significant loci (*p* = 2.51 × 10^−11^ to 1.25 × 10^−10^), located within or near genes involved in sodium ion transport (*commd3*), cell cycle regulation (*ccny*), protein ubiquitination (*commd10*), and nuclear export (*xpo7*). For combination 2 (TL, BL, and BD), MultiTrait detected highly significant SNPs within baz2a (chromatin remodeling) with a joint *p*-value of 3.91 × 10^−39^, and within fam81b (cell cycle regulation) with a joint *p*-value of 9.41 × 10^−81^; an additional locus near adamts18 (extracellular matrix degradation) was identified by GmatS_mv on chromosome 4. Combination 3 (HL, SnL, ED, PoL, and IW) yielded numerous significant loci (*p* = 3.78 × 10^−40^ to 9.54 × 10^−2^), with candidate genes including enah (cytoskeleton organization) and LOC118290135 (predicted GTPase activator). For combination 4 (TUL and CPD), MultiTrait identified SNPs within iqgap1 (signal transduction) and fam81b (cell cycle regulation), while GmatS_mv detected a locus near glra1 (neurotransmission) on chromosome 9 (Figure 5).

Of the 20 multi-trait SNPs, 16 (80%) were located within gene bodies, with the remaining four in intergenic regions 4–14 kb from the nearest gene. MultiTrait yielded the highest number of significant loci (9) and the smallest *p*-values, followed by GEMMA_mv (5) and GmatS_mv (3). To address potential multicollinearity among highly correlated traits (e.g., BW, TL, BL, and BD with r_g_ > 0.7), the multi-trait models incorporate a genetic covariance matrix that accounts for trait correlations, thereby mitigating the influence of collinearity on association statistics. Collectively, multi-trait joint analysis expanded upon single-trait GWAS results and revealed pleiotropic loci that contribute to the genetic architecture of growth traits in turbot.

**Figure 6:**
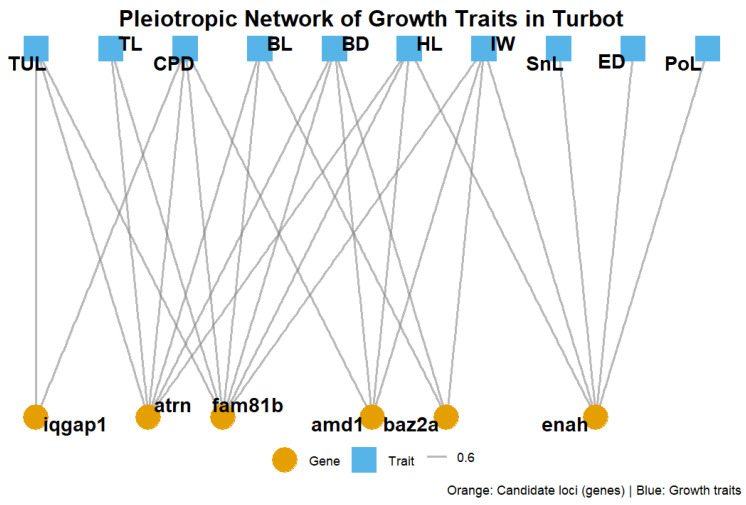
Pleiotropic network connecting significant candidate loci with 11 growth traits in turbot (*Scophthalmus maximus*). Orange nodes (left) represent candidate genes identified by single-trait or multi-trait GWAS; blue nodes (right) represent the 11 morphometric traits (BW, TL, BL, BD, TUL, HL, SnL, CPD, ED, PoL, IW). Each gray edge indicates a significant association (*p* < 1 × 10^−6^). Genes with five or more connections (e.g., *fam81b*, *atrn*) are highlighted as potential pleiotropic hubs, visually demonstrating the pervasive shared genetic regulation among growth traits.

To visualize the pleiotropic relationships among the identified loci and the 11 growth traits, we constructed a bipartite network diagram (Figure 6). This network clearly illustrates that certain genes, particularly fam81b and atrn, exhibit connections to six or more traits, reinforcing their role as core pleiotropic regulators. Notably, novel loci detected exclusively through multi-trait GWAS, such as iqgap1, also display strong connectivity to multiple body-size traits, supporting the effectiveness of our joint analysis approach in uncovering hidden pleiotropic signals. Overall, this graphical summary highlights the interconnected polygenic architecture underlying turbot growth.

### 2.6. KEGG Pathway Enrichment

**Figure 7:**
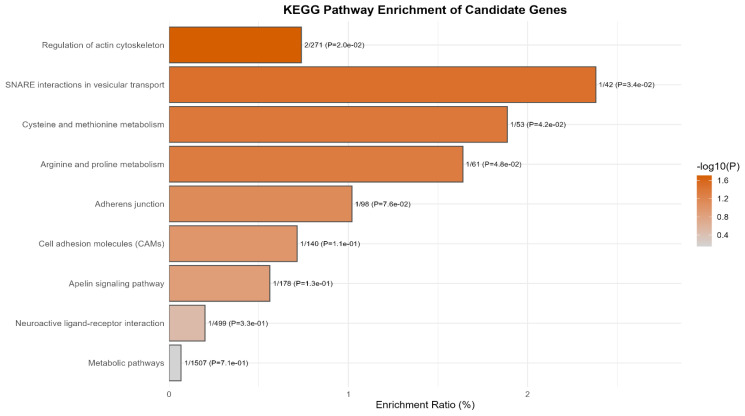
KEGG pathway enrichment analysis of candidate genes. A bar chart is used to show the enrichment ratios of significantly enriched pathways (number of matched genes/total genes in the pathway) for adjusted *p* < 0.05. Color gradient is −log_10_ (*p*-value).

**Figure 8:**
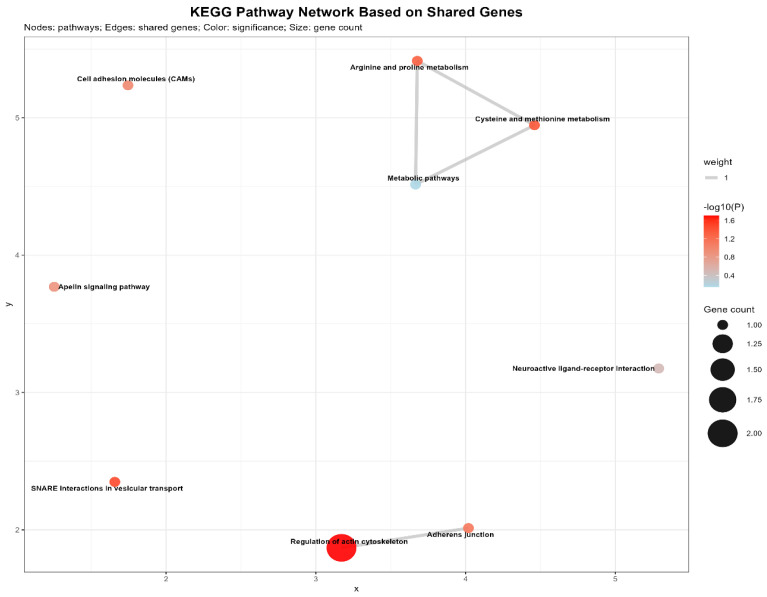
KEGG pathway network diagram based on shared candidate genes. Nodes are paths, the size of which is proportional to the number of matched genes, and the color intensity shows the significance level (−log_10_ (*p*-value)). Edges are the common genes of the pathways.

KEGG enrichment analysis of the 37 candidate genes revealed pathways showing nominal enrichment (uncorrected *p* < 0.05), among which only the “Regulation of actin cytoskeleton” pathway remained significant after multiple testing correction (adjusted *p* = 0.048) (Table 7; Figure 7). The other pathways, including SNARE interactions in vesicular transport, cysteine and methionine metabolism, and arginine and proline metabolism, showed suggestive trends but did not reach the adjusted significance threshold. Pathway network analysis further revealed that *amd1* connected three metabolic pathways, while *iqgap1* bridged cytoskeletal regulation with the adherens junction pathway (Figure 8), positioning both genes as hubs in the growth-regulatory network.

## 3. Discussion

Single-trait and multi-trait GWAS analyses were employed to systematically dissect the genetic architecture of 11 growth traits in turbot, identifying key candidate genes and pathways that may serve as targets for marker-assisted selection and functional studies.

### 3.1. Genetic Parameters of Growth Traits

Heritability estimates revealed that BW (h^2^ = 0.259) was the most heritable trait, consistent with estimates reported in olive flounder (0.21–0.35) [20]. Body size traits exhibited low to moderate heritability (0.115–0.132), indicating that phenotypic selection alone would yield slow genetic gains, necessitating the integration of molecular approaches. Head and eye traits showed very low heritability (≤0.05), similar to findings in rainbow trout [22]. Notably, ED and SnL displayed relatively large phenotypic coefficients of variation (CV = 24.87% and 18.72%) despite their low heritability, suggesting that most of their variation was environmentally driven [27,28].

Genetic correlation analysis revealed strong positive correlations between BW and most body size traits (*r*_g_ >> 0.7), supporting the feasibility of multi-trait synergistic improvement, consistent with reports in Atlantic salmon [23] and common carp [24]. The negative correlation between ED and BW (*r*_g_ = −0.047) has rarely been reported in fish and may reflect independent regulation due to developmental trade-offs or sensory system optimization. The relatively large standard errors for several heritability estimates (e.g., IW: 0.001 ± 0.147; ED: 0.004 ± 0.088) indicate limited estimation precision, and these values should be interpreted as preliminary approximations requiring validation in larger cohorts.

### 3.2. Candidate Genes from Single-TRAIT GWAS

SNP 19:16017254, repeatedly associated with six traits, maps to fam81b, a gene predicted to be involved in cell cycle regulation based on its human ortholog [29]. While this functional inference is speculative and lacks direct experimental validation in turbot, its pleiotropic effects could arise from its putative role in modulating cell proliferation and differentiation rates across multiple tissues, but this hypothesis requires functional testing. Similarly, SNP 15:2921091, located within atrn, is implicated in the melanocortin signaling pathway [9], which regulates energy homeostasis and appetite, suggesting that *atrn* may influence growth indirectly through metabolic efficiency.

*Amd1*, associated with multiple traits, is predicted to encode adenosylmethionine decarboxylase, a key enzyme in polyamine biosynthesis essential for DNA synthesis and cell division [30]. Other candidate genes, including *dlgap3* (synaptic signaling) and *slc6a1l* (GABA transport) [31], suggest that neural regulation may indirectly modulate growth through feeding behavior or neuroendocrine pathways, highlighting the interconnection between the nervous system and somatic growth.

It is important to interpret the observed multi-trait associations with caution. Given the strong genetic correlations among the growth traits examined (e.g., *r*_g_ > 0.7 between body weight and body size traits), a single locus associated with one core trait may appear to be associated with multiple traits merely due to statistical propagation of correlated phenotypes, rather than true biological pleiotropy. Distinguishing between genuine pleiotropy (one gene affects multiple independent biological pathways) and a correlated trait architecture (one gene affects a single underlying factor that influences multiple measured variables) requires additional approaches such as conditional GWAS or structural equation modeling. We acknowledge this limitation and therefore consider the reported pleiotropic loci as indicative of shared genetic regulation, warranting further fine-mapping and functional validation.

### 3.3. Novel Loci from Multi-Trait GWAS

None of the 20 multi-trait loci reached significance in single-trait GWAS, demonstrating the enhanced power of multi-trait analysis to detect pleiotropic variants that may be masked by trait-specific noise or small effect sizes. Among these, *baz2a*—identified in combination 2 (TL, BL, and BD)—encodes a bromodomain-adjacent zinc finger domain protein predicted to be involved in chromatin remodeling and transcriptional regulation. Chromatin remodelers such as BAZ2A can modulate gene expression programs during development by altering chromatin accessibility at growth-related loci, thereby potentially influencing body size traits. Its association with multiple body-size traits suggests that regulatory variation affecting chromatin architecture may contribute to growth variation, although direct evidence in turbot is currently lacking. *Iqgap1*, identified in combination 4 (TUL and CPD), functions in signal transduction and cytoskeletal organization, critical for muscle cell morphology and function. *Enah*, detected in combination 3 (HL, SnL, ED, PoL, and IW), modulates actin filaments and may influence head and eye morphology. The extremely small *p*-values for SNPs in baz2a and iqgap1 underscore their potential as core pleiotropic regulators. Collectively, these findings support the view that growth traits are associated with an interconnected polygenic network, with multi-trait GWAS offering a powerful lens to dissect shared genetic architecture. The substantial discrepancy among the three GWAS software packages (GEMMA: 45 significant SNPs; GmatS: 10; GCTA: 0) raises an important caveat regarding candidate gene prioritization. GEMMA’s greater sensitivity comes with a higher potential for false positives, whereas GCTA’s more conservative approach may miss genuine associations with modest effect sizes. To address this, we adopted a tiered confidence scheme: SNPs identified by GEMMA with extremely small *p*-values (*p* < 1 × 10^−20^) or those replicated across at least two software packages were designated as high-confidence candidates (*fam81b*, *atrn*, *amd1*); loci identified by only one package were treated as lower-confidence candidates requiring independent validation (Section 4.4).

### 3.4. Pathway Enrichment and Network Analysis

KEGG enrichment analysis identified the “Regulation of actin cytoskeleton” pathway as the most significantly enriched, involving *enah* and *iqgap1*. This pathway is fundamental to muscle development, myoblast fusion, and cell motility [8], directly impacting the measured growth traits. The SNARE pathway (*vti1a*), associated with vesicle transport and growth factor secretion [6], may influence nutrient uptake and hormonal signaling. Polyamine metabolic pathways (*amd1*) are essential for cell division and have been implicated in organ development [30]. As illustrated in the pathway network (Figure 7), *amd1* serves as a hub connecting three metabolic pathways, while *iqgap1* links cytoskeletal regulation to adherens junctions. Collectively, these findings provide preliminary, hypothesis-generating evidence for an interconnected polygenic network underlying turbot growth. The enrichment of the actin cytoskeleton pathway, though based on a limited set of candidate genes, offers a plausible direction for future functional investigations using gene editing or transcriptomic profiling.

### 3.5. Comparison with Previous Studies

Several genes identified in this study have been previously reported in GWAS of other fish species. For example, *wnt4* was associated with body shape traits in large yellow croaker *(Larimichthys crocea*) [21]; *pax7a* was linked to muscle development and growth in rainbow trout (*Oncorhynchus mykiss*) [22]; and *gnb3a* was associated with growth traits in common carp (*Cyprinus carpio*) [24]. While the direction of effect cannot be directly compared due to differences in trait definitions and population structures across studies, the recurrence of these genes across multiple teleost species supports the conservation of growth-regulatory mechanisms. Notably, novel associations of *fam81b*, *atrn*, and *iqgap1* with growth traits extend the known genetic map of fish growth. In particular, *iqgap1* has not been previously linked to fish growth, underscoring the value of multi-trait approaches for discovering new pleiotropic genes. These findings provide both validation of known loci and discovery of novel candidates for functional characterization.

### 3.6. Implications for Turbot Breeding

With a heritability of 0.259 for BW, genomic selection could accelerate genetic improvement over traditional pedigree-based approaches. The strong genetic correlations (*r*_g_ > 0.7) among body-size traits support the feasibility of indirect selection for multiple traits simultaneously. The candidate genes identified in this study—particularly the pleiotropic loci fam81b, iqgap1, enah, and baz2a—represent promising markers for marker-assisted selection (MAS). These markers warrant independent validation, and their functions should be verified through gene editing or expression analyses. SNPs located within gene bodies (e.g., in *amd1*) may exert direct functional effects and should be prioritized for fine-mapping and causal variant identification. Incorporating these markers into genomic selection models is expected to enhance predictive accuracy for growth traits and improve the efficiency of turbot breeding programs [29].

## 4. Materials and Methods

### 4.1. Experimental Population and Phenotyping

A total of 200 turbots from one production batch were taken from a farm in Yangjiapo Town, Binhai New Area, Tianjin. All fish were sampled at 1 year of age from the same production cohort. Individuals were randomly selected from pedigree records to reduce relatedness and do not choose full-sib or half-sib individuals. Eleven growth indices were evaluated according to the standard method; body weight (BW) were measured with an electronic scale that has a resolution of 0.01 g, and total length (TL), body length (BL), body depth (BD), trunk length (TUL), head length (HL), snout length (SnL), caudal peduncle depth (CPD), eye diameter (ED), post-orbital head length (PoL) and interorbital width (IW) were recorded with a digital caliper and have a resolution of 0.01 mm. Based on the functional correlations, the traits were grouped for multi-trait GWAS as follows: (1) BL, BD, BW; (2) TL, BL, BD; (3) HL, SnL, ED, PoL, IW; and (4) TUL, CPD. Before collection, anesthesia and euthanasia of the fish were carried out with MS-222 (150 mg/L), and then the tissue of the caudal fin was collected for genomic DNA extraction.

### 4.2. DNA Extraction, Sequencing and Genotyping

Genomic DNA was extracted from caudal fin tissue using the phenol–chloroform method. DNA integrity was assessed by agarose gel electrophoresis, and concentration was quantified using a fluorometer. Qualified samples were subjected to paired-end 150 bp sequencing on the BGI DNBSEQ platform (access date: 15 April 2026). The sequencing generated a total of approximately 1.2 Tb of clean data (an average of ~6 Gb per individual), with an average of ~20 million clean reads per individual. All samples had Q20 values > 95% and Q30 values > 87%, indicating high data quality.

Raw reads were filtered using SOAPnuke with parameters: -n 0.01 -l 20 -q 0.5 -adaMR 0.25 -polyX 50 -minReadLen 150. Clean reads were aligned to the turbot reference genome GCF_022379125.1 using BWA-MEM (version 0.7.17) with default parameters, and aligned reads were sorted and indexed using SAMtools (v1.10).

Variant calling was performed using GATK (version 4.1.8.0) via the HaplotypeCaller and GenotypeGVCFs workflow. Initial variants were filtered using BCFtools (version 1.22-8-g2d811c52+.) (DP < 10, MAF < 0.01), and missing genotypes were imputed using BEAGLE (version 4.1). Quality control was conducted using PLINK (version 2.0) to remove individuals with a genotype missing rate >5%, SNPs with a call rate < 95%, MAF < 0.01, and those deviating significantly from Hardy–Weinberg equilibrium (HWE *p* < 1 × 10^−6^). Finally, 177 individuals and 5,296,749 high-quality SNPs were retained for subsequent analyses.

### 4.3. Population Genetics and Heritability Estimation

To correct for population structure in subsequent GWAS analyses, we performed principal component analysis (PCA) using PLINK (version 2.0) on a set of LD-pruned SNPs (window size: 50 kb, step: 5 SNPs, r^2^ threshold: 0.2). The first ten PCs were retained as covariates in the GWAS models (Section 4.4). The indices of genetic diversity, i.e., observed heterozygosity (Ho), expected heterozygosity (He) and inbreeding coefficient (F), were obtained with VCFtools and PLINK. GCTA (version 1.94.1) was employed to construct the genomic relationship matrix (GRM) [26]. Heritability was estimated using REML implemented in GCTA and genetic correlation between traits by Bivariate Linear Mixed Models was used.

### 4.4. Genome-Wide Association Study

Single-trait GWAS was carried out by the three software packages: GEMMA (version 0.98.5), GCTA (v1.94.1) and GmatS (version 1.01). Age and the first few principal components were added as covariates to correct for population structure. Four trait groups were studied with multi-trait GWAS by means of GEMMA_mv, the R package MultiTrait and GmatS_mv. A genome-wide significance threshold of *p* < 1 × 10^−6^ was applied to declare association. This threshold, while less stringent than a Bonferroni-corrected threshold of 0.05/5.3 × 10^6^ ≈ 9.4 × 10^−9^, was chosen to balance sensitivity and specificity given the moderate sample size and the high linkage disequilibrium among markers typical of selectively bred aquaculture populations, and is consistent with previous GWAS in teleosts [20,25]. The top five most significant SNPs for all the features were selected to be investigated further. Manhattan and Q-Q plots were drawn in R (version 4.5.2) [27,32]. To ensure that the multi-trait GWAS identified new loci not covered by those in the single-trait analyses, we did not include any SNPs that had already reached the significance level (*p* < 1 × 10^−6^) in any one of the single-trait GWASs. Only the SNPs that were positively associated with many traits were chosen as multi-trait specific loci.

It is noteworthy that the three GWAS packages yielded different numbers of significant SNPs (GEMMA: 45; GmatS: 10; GCTA: 0) among the top five per trait. This discrepancy likely reflects differences in their underlying algorithms: GEMMA uses an efficient mixed-model association (EMMA) algorithm with a full kinship matrix; GmatS employs a score-based test with a compressed mixed model; and GCTA’s MLM-based approach is known to be more conservative, particularly with small sample sizes. To minimize false positives and ensure robustness, we prioritized SNPs identified by GEMMA with extreme significance (*p* < 1 × 10^−20^) or those replicated across at least two software packages. Importantly, the core pleiotropic signals (fam81b, atrn, amd1) were consistently detected by GEMMA and, in part, by GmatS, supporting their reliability.

### 4.5. Candidate Gene Annotation and Pathway Enrichment

The genomic region of the candidate was defined as the area 50 kb away from the top SNP in either direction [33]. Genes in the above regions were obtained from the turbot reference genome annotation (GCF_022379125.1). Candidate genes were mapped to zebrafish orthologs and then sent to KOBAS for KEGG pathway enrichment analysis. To determine if it is an increase, a hypergeometric test was conducted, and then the Benjamini–Hochberg method was used to correct the *p*-values (adj *p* < 0.05). Bar plots and pathway networks were drawn with ggplot2 in R [34].

## 5. Limitations

Several limitations of this study should be acknowledged. (1) Limited statistical power and potential overestimation of effects: The sample size (*n* = 177) is relatively modest for a genome-wide association study of complex quantitative traits. Despite the use of a stringent significance threshold (*p* < 1 × 10^−6^) and three independent software packages to reduce false positives, we acknowledge that the effect sizes of the identified SNPs are likely subject to the “winner’s curse” (i.e., overestimation in small discovery samples). Therefore, these genetic parameters and effect estimates should be viewed as upper-bound approximations requiring validation in expanded cohorts. (2) Functional annotation relied on zebrafish orthology, and the turbot genome annotation remains incomplete. (3) KEGG enrichment was performed only on known pathways, potentially missing fish-specific regulatory mechanisms. (4) Functional validation of the candidate genes is lacking. (5) Lack of independent replication: All individuals were sampled from a single commercial farm in Tianjin. The absence of an independent validation population (e.g., from different geographical locations or generations) means that the generalizability of our findings remains unknown. The loci identified here should therefore be considered candidate QTLs rather than validated markers for immediate application in breeding.

## 6. Conclusions

In this study, we performed single-trait and multi-trait GWAS to dissect the genetic architecture of 11 growth traits in 177 turbot individuals. Our findings demonstrate that body weight is the most heritable trait (h^2^ = 0.259) and is strongly genetically correlated with most body size traits (*r_g_* > 0.7), supporting the feasibility of multi-trait synergistic selection.

A key discovery of this study is the pervasive pleiotropy underlying turbot growth. Single-trait GWAS identified two pleiotropic loci—*fam81b* and *atrn*—repeatedly associated with six body size traits, while multi-trait GWAS uncovered 20 novel loci, including *iqgap1*, *enah*, and *baz2a*, that were not detectable in single-trait analyses. These findings highlight the power of multi-trait approaches in revealing hidden pleiotropic signals.

KEGG enrichment further implicated the actin cytoskeleton pathway and polyamine metabolism in growth regulation, with *amd1* serving as a hub connecting multiple metabolic pathways.

Collectively, these results provide robust molecular markers for marker-assisted selection and lay a foundation for genomic selection in turbot breeding programs. Functional validation of the candidate genes in future studies will be essential to confirm their roles in growth regulation.

## Figures and Tables

**Table 1:** Genetic correlation coefficients of the growth traits calculated by R (v4.5.2).

	TL	BL	BD	TUL	HL	SnL	CPD	ED	PoL	IW	BW
TL	1	0.872	0.872	0.837	0.593	0.509	0.694	0.2	0.432	0.498	0.842
BL		1	0.789	0.786	0.568	0.165	0.741	0.349	0.471	0.374	0.766
BD			1	0.735	0.504	0.395	0.713	0.105	0.404	0.457	0.864
TUL				1	0.293	0.467	0.585	0.151	0.087	0.386	0.674
HL					1	0.381	0.394	0.261	0.776	0.435	0.468
SnL						1	0.103	−0.232	0.076	0.301	0.342
CPD							1	0.184	0.317	0.287	0.73
ED								1	0.175	0.119	−0.047
PoL									1	0.396	0.416
IW										1	0.434
BW											1

Note: Table 1 shows the genetic correlations (below diagonal) of 11 growth-related traits in turbot. The values in bold are positively correlated with |*r*_g_| > 0.7. Bivariate linear mixed models were used to calculate all the correlations in GCTA.

**Table 2:** Description statistics and heritability estimates of growth traits in Turbot (*Scophthalmus maximus*).

Trait	N_Individuals	Mean	Sd	CV (%)	h^2^	Se
BW	177	70.613	15.486	21.93	0.259	0.158
TL	177	15.282	0.942	6.16	0.121	0.114
BL	177	11.893	0.831	6.99	0.118	0.142
BD	177	8.791	0.642	7.30	0.132	0.148
TUL	177	7.371	0.611	8.28	0.115	0.124
HL	177	4.208	0.430	10.22	0.024	0.106
SnL	177	1.063	0.199	18.72	0.052	0.089
CPD	177	1.732	0.145	8.37	0.005	0.103
ED	177	0.575	0.143	24.87	0.004	0.088
PoL	177	2.597	0.347	13.36	0.004	0.089
IW	177	1.263	0.130	10.29	0.001	0.147

**Table 3:** PCA results of 177 turbot individuals based on 5.29 million high-quality SNPs.

Principal Component	Eigenvalue	Variance Explained (%)	Cumulative (%)
PC1	6.901	19.39	19.39
PC2	5.397	15.17	34.56
PC3	3.601	10.12	44.68
PC4	3.378	9.49	54.17
PC5	3.144	8.83	63.00
PC6	2.828	7.95	70.95
PC7	2.686	7.55	78.5
PC8	2.626	7.38	85.88
PC9	2.528	7.11	92.98
PC10	2.497	7.02	100

**Table 4:** Genetic diversity indices of all sampled individuals (*n* = 177).

Parameter	Value
Number of individuals	177
Mean observed heterozygosity (Ho)	0.1818
Mean expected heterozygosity (He)	0.1979
Mean inbreeding coefficient (F)	0.0813
Ho range	0.0934–0.2229
He range	0.1979–0.1980
F range	−0.126–0.0813

Note: The narrow range of He reflects the homogeneous genetic background of this farmed population, which is typical of selectively bred aquaculture stocks.

**Table 5:** Summary of significant SNPs and candidate genes for the 11 growth-related traits of turbot (*Scophthalmus maximus*) identified by single-trait GWAS.

Trait	Software	Chr	Pos	*p*_Value	Nearest_Gene	Gene Function	Distance_to_Gene
BD	GEMMA	19	16,017,254	4.81 × 10^−26^	fam81b	Predicted to be involved in cell cycle regulation. Orthologous to human FAM81B (family with sequence similarity 81 member B).	0
BD	GEMMA	18	1,506,245	5.69 × 10^−26^	amd1	Enables adenosylmethionine decarboxylase activity. Involved in polyamine biosynthesis. Orthologous to human AMD1 (adenosylmethionine decarboxylase 1).	0
BD	GEMMA	3	2,415,956	5.88 × 10^−26^	baz2a	Predicted to enable chromatin binding and DNA-binding transcription factor activity. Involved in chromatin remodeling and transcriptional regulation. Orthologous to human BAZ2A (bromodomain adjacent to zinc finger domain 2A).	20,659
BD	GEMMA	15	2,921,091	6.02 × 10^−26^	atrn	Enables attractin activity. Involved in melanocortin signaling pathway and immune response. Orthologous to human ATRN (attractin).	0
BD	GEMMA	15	27,552,577	6.02 × 10^−26^	LOC124849372	Predicted to enable ubiquitin protein ligase activity. Orthologous to human RNF213 (ring finger protein 213).	4,828,159
BL	GEMMA	19	16,017,254	3.07 × 10^−28^	fam81b	Predicted to be involved in cell cycle regulation. Orthologous to human FAM81B (family with sequence similarity 81 member B).	0
BL	GEMMA	3	2,415,956	4.20 × 10^−28^	baz2a	Predicted to enable chromatin binding and DNA-binding transcription factor activity. Involved in chromatin remodeling and transcriptional regulation. Orthologous to human BAZ2A (bromodomain adjacent to zinc finger domain 2A).	20,659
BL	GEMMA	15	2,921,091	4.28 × 10^−28^	atrn	Enables attractin activity. Involved in melanocortin signaling pathway and immune response. Orthologous to human ATRN (attractin).	0
BL	GEMMA	15	27,552,577	4.28 × 10^−28^	LOC124849372	Predicted to enable ubiquitin protein ligase activity. Orthologous to human RNF213 (ring finger protein 213).	4,828,159
BL	GEMMA	16	11,535,673	4.28 × 10^−28^	LOC118287997	Predicted to enable GTPase activator activity. Orthologous to human ARHGAP42 (Rho GTPase activating protein 42).	1439
BW	GmatS	7	9,954,324	1.25 × 10^−8^	plxnb2b	Predicted to enable semaphorin receptor activity. Involved in axon guidance and cell migration. Orthologous to human PLXNB2 (plexin B2).	0
BW	GmatS	9	1,174,249	3.62 × 10^−8^	LOC118319474	Predicted to enable DNA-binding transcription factor activity. Orthologous to human HHEX (hematopoietically expressed homeobox).	119,238
BW	GmatS	22	2,352,948	2.26 × 10^−7^	dlgap3	Predicted to enable scaffold protein activity. Involved in synaptic signaling and neurotransmitter receptor clustering. Orthologous to human DLGAP3 (DLG associated protein 3).	0
BW	GmatS	22	2,352,902	2.48 × 10^−7^	dlgap3	Predicted to enable scaffold protein activity. Involved in synaptic signaling and neurotransmitter receptor clustering. Orthologous to human DLGAP3 (DLG associated protein 3).	0
BW	GEMMA	20	23,447,437	2.91 × 10^−7^	LOC124849736	Predicted to enable ATP binding and helicase activity. Orthologous to human CHD8 (chromodomain helicase DNA binding protein 8).	5,004,976
CPD	GEMMA	19	16,017,254	4.82 × 10^−20^	fam81b	Predicted to be involved in cell cycle regulation. Orthologous to human FAM81B (family with sequence similarity 81 member B).	0
CPD	GEMMA	18	1,506,245	5.07 × 10^−20^	amd1	Enables adenosylmethionine decarboxylase activity. Involved in polyamine biosynthesis. Orthologous to human AMD1 (adenosylmethionine decarboxylase 1).	0
CPD	GEMMA	15	2,921,091	5.55 × 10^−20^	atrn	Enables attractin activity. Involved in melanocortin signaling pathway and immune response. Orthologous to human ATRN (attractin).	0
CPD	GEMMA	15	27,552,577	5.55 × 10^−20^	LOC124849372	Predicted to enable ubiquitin protein ligase activity. Orthologous to human RNF213 (ring finger protein 213).	4,828,159
CPD	GEMMA	16	11,535,673	5.55 × 10^−20^	LOC118287997	Predicted to enable GTPase activator activity. Orthologous to human ARHGAP42 (Rho GTPase activating protein 42).	1439
ED	GmatS	2	16,257,256	7.38 × 10^−8^	LOC118300455	Predicted to enable serine-type endopeptidase inhibitor activity. Orthologous to human SERPINA5 (serpin family A member 5).	0
ED	GmatS	6	23,352,525	9.62 × 10^−8^	wnt4	Enables Wnt-protein binding activity. Involved in cell signaling, embryonic development, and tissue homeostasis. Orthologous to human WNT4 (Wnt family member 4).	4344
ED	GmatS	1	27,788,564	1.00 × 10^−7^	LOC118301237	Predicted to enable RNA binding. Orthologous to human RBM3 (RNA binding motif protein 3).	0
ED	GmatS	2	19,434,538	3.70 × 10^−7^	trnae-uuc_1	Transfer RNA for glutamic acid (anticodon UUC). Involved in protein synthesis.	21,231
ED	GmatS	12	16,185,086	4.43 × 10^−7^	mdfic	Predicted to enable DNA-binding transcription factor activity. Involved in cardiac development. Orthologous to human MDFIC (MyoD family inhibitor domain containing).	8062
HL	GEMMA	20	2,595,381	1.72 × 10^−16^	tprn	Predicted to enable tautomerase activity. Involved in auditory system development. Orthologous to human TPRN (taperin).	0
HL	GEMMA	19	16,017,254	1.21 × 10^−15^	fam81b	Predicted to be involved in cell cycle regulation. Orthologous to human FAM81B (family with sequence similarity 81 member B).	0
HL	GEMMA	18	1,506,245	1.27 × 10^−15^	amd1	Enables adenosylmethionine decarboxylase activity. Involved in polyamine biosynthesis. Orthologous to human AMD1 (adenosylmethionine decarboxylase 1).	0
HL	GEMMA	15	2,921,091	1.38 × 10^−15^	atrn	Enables attractin activity. Involved in melanocortin signaling pathway and immune response. Orthologous to human ATRN (attractin).	0
HL	GEMMA	15	27,552,577	1.38 × 10^−15^	LOC124849372	Predicted to enable ubiquitin protein ligase activity. Orthologous to human RNF213 (ring finger protein 213).	4,828,159
IW	GEMMA	21	24,317,263	5.35 × 10^−15^	LOC118291400	Predicted to enable protein kinase activity. Orthologous to human PRKCE (protein kinase C epsilon).	8,300,901
IW	GEMMA	21	24,317,264	5.35 × 10^−15^	LOC118291400	Predicted to enable protein kinase activity. Orthologous to human PRKCE (protein kinase C epsilon).	8,300,902
IW	GEMMA	18	1,506,245	1.26 × 10^−14^	amd1	Enables adenosylmethionine decarboxylase activity. Involved in polyamine biosynthesis. Orthologous to human AMD1 (adenosylmethionine decarboxylase 1).	0
IW	GEMMA	19	16,017,254	1.28 × 10^−14^	fam81b	Predicted to be involved in cell cycle regulation. Orthologous to human FAM81B (family with sequence similarity 81 member B).	0
IW	GEMMA	3	2,415,956	1.32 × 10^−14^	baz2a	Predicted to enable chromatin binding and DNA-binding transcription factor activity. Involved in chromatin remodeling and transcriptional regulation. Orthologous to human BAZ2A (bromodomain adjacent to zinc finger domain 2A).	20,659
PoL	GmatS	12	19,202,523	4.25 × 10^−16^	slc6a1l	Predicted to enable neurotransmitter:sodium symporter activity. Involved in GABA uptake. Orthologous to human SLC6A1 (solute carrier family 6 member 1).	0
PoL	GmatS	12	19,202,527	4.25 × 10^−16^	slc6a1l	Predicted to enable neurotransmitter:sodium symporter activity. Involved in GABA uptake. Orthologous to human SLC6A1 (solute carrier family 6 member 1).	0
PoL	GmatS	1	9,627,221	4.89 × 10^−16^	znf1035	Predicted to enable DNA-binding transcription factor activity. Involved in transcriptional regulation. Orthologous to human ZNF103 (zinc finger protein 103).	0
PoL	GmatS	1	14,216,872	4.89 × 10^−16^	gnb3a	Predicted to enable GTPase activity. Involved in G-protein coupled receptor signaling. Orthologous to human GNB3 (G protein subunit beta 3).	0
PoL	GmatS	1	14,216,876	4.89 × 10^−16^	gnb3a	Predicted to enable GTPase activity. Involved in G-protein coupled receptor signaling. Orthologous to human GNB3 (G protein subunit beta 3).	0
SnL	GmatS	6	10,503,603	5.48 × 10^−8^	pax7a	Enables DNA-binding transcription factor activity. Involved in muscle development and neurogenesis. Orthologous to human PAX7 (paired box 7).	0
SnL	GEMMA	17	25,026,979	1.14 × 10^−7^	scn4ab	Predicted to enable voltage-gated sodium channel activity. Involved in action potential propagation. Orthologous to human SCN4A (sodium voltage-gated channel alpha subunit 4).	4,269,862
SnL	GmatS	14	3,487,145	1.35 × 10^−7^	xpo4	Predicted to enable nuclear export signal receptor activity. Involved in protein export from nucleus. Orthologous to human XPO4 (exportin 4).	0
SnL	GmatS	1	24,385,902	1.76 × 10^−7^	nlgn4xa	Predicted to enable neuroligin activity. Involved in synapse formation and function. Orthologous to human NLGN4X (neuroligin 4 X-linked).	0
SnL	GmatS	1	24,385,887	2.43 × 10^−7^	nlgn4xa	Predicted to enable neuroligin activity. Involved in synapse formation and function. Orthologous to human NLGN4X (neuroligin 4 X-linked).	0
TL	GEMMA	19	16,017,254	3.35 × 10^−32^	fam81b	Predicted to be involved in cell cycle regulation. Orthologous to human FAM81B (family with sequence similarity 81 member B).	0
TL	GEMMA	15	2,921,091	4.62 × 10^−32^	atrn	Enables attractin activity. Involved in melanocortin signaling pathway and immune response. Orthologous to human ATRN (attractin).	0
TL	GEMMA	15	27552,577	4.62 × 10^−32^	LOC124849372	Predicted to enable ubiquitin protein ligase activity. Orthologous to human RNF213 (ring finger protein 213).	4,828,159
TL	GEMMA	16	11,535,673	4.62 × 10^−32^	LOC118287997	Predicted to enable GTPase activator activity. Orthologous to human ARHGAP42 (Rho GTPase activating protein 42).	1439
TL	GEMMA	16	14,169,682	4.62 × 10^−32^	vti1a	Predicted to enable SNARE binding activity. Involved in vesicle-mediated transport. Orthologous to human VTI1A (vesicle transport through interaction with t-SNAREs 1A).	0
TUL	GEMMA	19	16,017,254	6.03 × 10^−22^	fam81b	Predicted to be involved in cell cycle regulation. Orthologous to human FAM81B (family with sequence similarity 81 member B).	0
TUL	GEMMA	15	2,921,091	7.19 × 10^−22^	atrn	Enables attractin activity. Involved in melanocortin signaling pathway and immune response. Orthologous to human ATRN (attractin).	0
TUL	GEMMA	15	27,552,577	7.19 × 10^−22^	LOC124849372	Predicted to enable ubiquitin protein ligase activity. Orthologous to human RNF213 (ring finger protein 213).	4,828,159
TUL	GEMMA	16	11,535,673	7.19 × 10^−22^	LOC118287997	Predicted to enable GTPase activator activity. Orthologous to human ARHGAP42 (Rho GTPase activating protein 42).	1439
TUL	GEMMA	16	14,169,682	7.19 × 10^−22^	vti1a	Predicted to enable SNARE binding activity. Involved in vesicle-mediated transport. Orthologous to human VTI1A (vesicle transport through interaction with t-SNAREs 1A).	0

**Table 6:** Top SNPs and candidate genes from multi-trait GWAS.

Trait Combination	Method	Chr	Pos (bp)	*p*_Value	Nearest_Gene	Gene Function	Distance to Gene
BL, BW, BD	GEMMA_mv	21	5,106,719	2.51 × 10^−11^	commd3	Predicted to act upstream of or within sodium ion transport. Orthologous to human COMMD3 (COMM domain containing 3).	0
BL, BW, BD	GEMMA_mv	21	8,857,266	3.91 × 10^−11^	ccny	Predicted to enable cyclin-dependent protein kinase activity. Involved in regulation of cell cycle. Orthologous to human CCNY (cyclin Y).	0
BL, BW, BD	GEMMA_mv	19	8,288,631	5.36 × 10^−11^	commd10	Predicted to be involved in protein ubiquitination and NF-κB signaling. Orthologous to human COMMD10 (COMM domain containing 10).	0
BL, BW, BD	GEMMA_mv	20	415,685	1.25 × 10^−10^	xpo7	Predicted to enable nuclear export signal receptor activity. Involved in protein export from nucleus. Orthologous to human XPO7 (exportin 7).	4768
BL, BW, BD	GEMMA_mv	20	415,689	1.25 × 10^−10^	xpo7	Predicted to enable nuclear export signal receptor activity. Involved in protein export from nucleus. Orthologous to human XPO7 (exportin 7).	4772
TL, BL, BD	MultiTrait	19	16,017,254	9.41 × 10^−81^	fam81b	Predicted to be involved in cell cycle regulation. Orthologous to human FAM81B (family with sequence similarity 81 member B)	0
TL, BL, BD	MultiTrait	3	2,415,956	3.91 × 10^−39^	baz2a	Predicted to enable chromatin binding and DNA-binding transcription factor activity. Involved in chromatin remodeling and transcriptional regulation. Orthologous to human BAZ2A (bromodomain adjacent to zinc finger domain 2A).	546
TL, BL, BD	GmatS_mv	4	23,425,698	8.68 × 10^−8^	adamts18	Predicted to enable metalloendopeptidase activity. Involved in extracellular matrix organization and degradation. Orthologous to human ADAMTS18 (ADAM metallopeptidase with thrombospondin type 1 motif 18).	14,188
HL, SnL, ED, PoL, IW	MultiTrait	6	2,595,381	3.78 × 10^−40^	LOC118308749	Predicted to enable protein binding. Orthologous to human FAM171A1 (family with sequence similarity 171 member A1).	0
HL, SnL, ED, PoL, IW	MultiTrait	18	3,771,168	2.05 × 10^−39^	enah	enah	0
HL, SnL, ED, PoL, IW	GmatS_mv	18	13,430,185	9.54 × 10^−23^	LOC118290135	Predicted to enable GTPase activator activity. Orthologous to human RGS6 (regulator of G protein signaling 6).	0
TUL, CPD	MultiTrait	19	16,017,254	2.75 × 10^−39^	fam81b	Predicted to be involved in cell cycle regulation. Orthologous to human FAM81B (family with sequence similarity 81 member B)	0
TUL, CPD	MultiTrait	4	1,506,245	3.66 × 10^−39^	iqgap1	Predicted to enable calmodulin binding and GTPase activator activity. Involved in cell signaling and cytoskeleton organization. Orthologous to human IQGAP1 (IQ motif containing GTPase activating protein 1).	0
TUL, CPD	GmatS_mv	9	24,910,413	1.57 × 10^−8^	glra1	Predicted to enable glycinergic chloride channel activity. Involved in neurotransmission and inhibitory synapse function. Orthologous to human GLRA1 (glycine receptor alpha 1).	4335

Note: All SNPs in this table reached the genome-wide significance threshold (*p* < 1 × 10^−6^) in multi-trait GWAS but did not reach significance in any single-trait analysis (*p* > 1 × 10^−6^). New pleiotropic loci that have been found in multi-trait analysis are being named.

**Table 7:** Detailed information of the nine KEGG pathways enriched by candidate genes in Turbot (*Scophthalmus maximus*).

Rank	Pathway	ID	Input_Genes	Total_Genes	Enrichment_Ratio	*p*_Value	Corrected_*p*	Genes	Significant
1	Regulation of actin cytoskeleton	dre04810	2	271	0.74%	2.00 × 10^−2^	4.80 × 10^−2^	enah iqgap1	Yes
2	SNARE interactions in vesicular transport	dre04130	1	42	2.38%	3.40 × 10^−2^	6.00 × 10^−2^	vti1a	Yes
3	Cysteine and methionine metabolism	dre00270	1	53	1.89%	4.20 × 10^−2^	7.00 × 10^−2^	amd1	Yes
4	Arginine and proline metabolism	dre00330	1	61	1.64%	4.80 × 10^−2^	7.60 × 10^−2^	amd1	Yes
5	Adherens junction	dre04520	1	98	1.02%	7.60 × 10^−2^	1.00 × 10^−1^	iqgap1	No
6	Cell adhesion molecules (CAMs)	dre04514	1	140	0.71%	1.10 × 10^−1^	1.30 × 10^−1^	nlgn4xa	No
7	Apelin signaling pathway	dre04371	1	178	0.56%	1.30 × 10^−1^	1.60 × 10^−1^	gnb3a	No
8	Neuroactive ligand-receptor interaction	dre04080	1	499	0.20%	3.30 × 10^−1^	3.50 × 10^−1^	glra1	No
9	Metabolic pathways	dre01100	1	1507	0.07%	7.10 × 10^−1^	7.10 × 10^−1^	amd1	No

## Data Availability

The raw data supporting the conclusions of this article will be made available by the authors on request.
